# A Review of Multimodal Medical Image Fusion Techniques

**DOI:** 10.1155/2020/8279342

**Published:** 2020-04-23

**Authors:** Bing Huang, Feng Yang, Mengxiao Yin, Xiaoying Mo, Cheng Zhong

**Affiliations:** ^1^School of Computer and Electronics and Information, Guangxi University, Nanning, Guangxi 530004, China; ^2^Guangxi Key Laboratory of Multimedia Communications Network Technology, China

## Abstract

The medical image fusion is the process of coalescing multiple images from multiple imaging modalities to obtain a fused image with a large amount of information for increasing the clinical applicability of medical images. In this paper, we attempt to give an overview of multimodal medical image fusion methods, putting emphasis on the most recent advances in the domain based on (1) the current fusion methods, including based on deep learning, (2) imaging modalities of medical image fusion, and (3) performance analysis of medical image fusion on mainly data set. Finally, the conclusion of this paper is that the current multimodal medical image fusion research results are more significant and the development trend is on the rise but with many challenges in the research field.

## 1. Introduction

Since the emergence of image fusion in 1985, image information fusion has developed rapidly on military and civilian fields, especially image fusion of infrared and visible light, material analysis, remote sensing image fusion, multifocus image, and medical image fusion. Imaging technology plays an important role in medical diagnosis. The limited information provided by single modal medical images cannot meet the need of clinical diagnosis which requires a large amount of information, making medical image fusion research become a hot field. Medical image fusion can be divided into single-mode fusion and multimodal fusion. Due to the limitation of the information presented by single-modal fusion, there are many researchers engaging in the study of multimodal fusion.

In the field of medical image, imaging techniques such as Computed Tomography (CT), Magnetic Resonance Imaging (MRI), Positron Emission Tomography (PET), and Single-Photon Emission Computed Tomography (SPECT) have provided clinicians with information of the human body's structural characteristics, soft tissue, and so on. Different imaging methods keep different characteristics, and different sensors obtain different imaging information of the same part. The purpose of the fusion is to obtain better contrast, fusion quality, and perceived experience. The result of the fusion should meet the following conditions: (a) the fused image should retain the information of source images completely; (b) the fused image should not produce any synthetic information, such as artifacts; and (c) bad states should be avoided, such as misregistration and noise [1].

Traditional medical image fusion methods are divided into spatial domain and transform domain. The medical image fusion methods based on spatial domain were the hotspot of the earliest research. The typical methods are principal analysis and HIS. However, spatial domain technology produces spectral distortion and spatial distortion of fused images [2]. For better fusion effects, researchers turn their research focus to the transform domain. It transforms the source image into the frequency domain or other domains to fuse them and then performs reconstruction operations. The fusion process is divided into four levels, namely, signal, feature, symbol, and pixel level [3]. The pixel level is widely used nowadays, and its typical representatives include contour transformation, discrete wavelet transform, and pyramid transform. The transform domain-based method has the advantages of good structure and avoiding distortion, but it also generates noise during the fusion processing. Therefore, denoising is also a challenge for image fusion [4, 5]. From the papers of the past two years, it can be seen that there is almost no method for the proposed fusion algorithm to using spatial domain alone. However, there are many new methods that combine spatial domain methods with transform domain, such as PCA-DWT [6]. With the advent of the deep learning boom, a medical image fusion method based on deep learning emerged in 2017. In recent years, convolutional neural network (CNN), recurrent neural network (RNN), U-Net network, GAN, and other deep learning models have been widely used in medical image registration and segmentation, while only CNN and U-Net network have been applied into medical image fusion. Convolutional neural network is a kind of neural network for image process, which is composed of convolutional layer, pooling layer, and fully connected layer. Deep learning framework for medical image fusion includes Caffe, Tensorflow, MatConvNet, and the like. At present, U-Net network is found to be trained on Pytorch deep learning framework.

**Figure 1:**
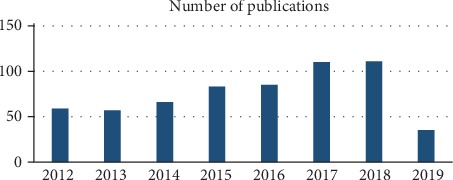
Number of published papers with of medical image fusion.

From 2012 to August 2019, medical image fusion technology has been developed significantly, as shown in Figure 1: the number of published papers of medical image fusion has remarkably boomed in the recent years (medical image fusion publications were counted by web of science, from 2012 to August 2019).

The purpose of this review is to summarize the research progresses and future development of this field combining with scientific papers about medical image fusion in recent years. This paper is mainly divided into the following sections:

1. Introduction to the current fusion methods
2. Mode of multimodal fusion
3. Comparing the data of different medical image fusion methods in the same database with the same evaluation index
4. Discussing the challenges of medical image fusion methods and future research trends

## 2. Fusion Methods

This chapter introduces the methods of medical image fusion from three aspects, the fusion method based on spatial domain, the fusion method based on transform domain, and the fusion method based on deep learning.

### 2.1. Spatial Domain

The medical image fusion technology based on spatial domain is the hot topic in early research. Its fusion technology is simple, and the fusion rules can be directly applied to the source image pixels to obtain the merged image. The fusion methods of spatial domain include the high-pass filtering method, the principal component analysis method, the saturation method of hue intensity, the average method, the maximum selection method, the minimum selection method, and the Brovey method. Due to the spectral distortion and spatial distortion in the fused image of the spatial domain, the heat of research in the spatial domain of the medical image fusion method is gradually decreasing in recent years. Researchers often use spatial domain fusion strategies as a part of the transformation domain to form new research methods. We will only briefly introduce the IHS method with a high usage value as below.

#### 2.1.1. Fusion Method Based on IHS Domain

The IHS model proposed by an American scientist Munsell explains the characteristics of the human visual system. It has two characteristics: (1) the intensity component has nothing to do with the color information of the image; (2) hue and saturation components are closely related to the way people perceive color. Therefore, researchers often use this model to solve the color problem in the image fusion process, especially the fusion of PET/SPECT images with color information.

**Figure 2:**
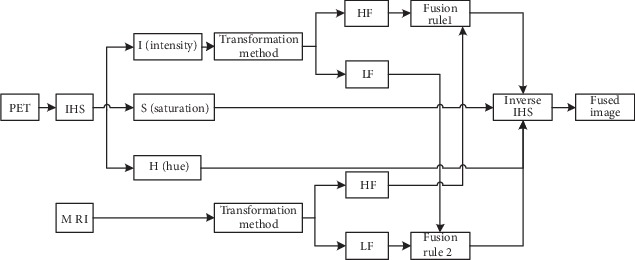
Framework diagram based on the IHS domain fusion method.

Chen [7] combined the IHS model with Log-Gabor transform to propose a new method about the fusion of MRI and PET and decomposed the PET image with IHS to obtain the three basic characteristics of hue (H), saturation (S), and intensity (I). The component intensity represents the brightness of the image, so the intensity components of the MRI and PET images are decomposed by the Log-Gabor transform consisting of the logarithmic transformation of the Gabor filter to obtain the high-frequency subbands and the low-frequency subbands. Fusion of high-frequency subbands comes with maximum selection; fusion of low-frequency subbands comes with a new method based on two-level fusion of visibility measurement and weighted average rule. The inverse Log-Gabor transformed component and the original hue and saturation components are inversely HIS to obtain a fused image. It can effectively preserve the structures and details of the source image and reduce the color distortion. This method is superior to the existing IHS+FT method in visual perception. Haddadpour et al. [8] proposed a new fusion method which combining the IHS method with the two-dimensional Hilbert transform. The method [9] introduces the concept of BEMD when merging high- and low-frequency subbands. BEMD is called bidirectional empirical mode decomposition and is extended by empirical mode decomposition. It is widely used in biomedicine field because of its envelope surface. The algorithm has no obvious distortion and is superior to the PCA and wavelet algorithms in terms of contrast and color intensity. Its disadvantage is that the information entropy (EN) is relatively low.  Figure 2 shows the framework of the IHS domain fusion method based on the fusion of MRI and PET images.

In order to achieve better results, different researchers tend to study the decomposition transform, such as DST and Log-Gabor transform. And they study fusion algorithm about the decomposition transform, such as SR algorithm and maximum selection algorithm.

### 2.2. Transform Domain

The medical image fusion methods of the transform domain are mostly based on the multiscale transform (MST) theory, and they are also the hotspots of research in recent years. The MST-based fusion method is generally divided into three steps: decomposition, fusion, and reconstruction. The medical image fusion method based on transform domain is transforming the source image from time domain to frequency domain or other domains to obtain the low-frequency coefficient and high-frequency coefficient. This section focuses on three most commonly used transformations in medical image fusion methods: nonsubsampled contourlet transform, nonsubsampled shearlet transform, and discrete wavelet transform.

#### 2.2.1. Fusion Based on Nonsubsampled Contourlet Transform (NSCT)

The contourlet transform is multiscale which is proposed by Do et al. [10]. It is suitable for constructing multiresolution and multidirectional situations and has advantages in smoothness processing. However, it does not have translation invariance, and it is easy to generate pseudo-Gibbs phenomenon (artifact) near the singular point of the reconstructed image, resulting in image distortion, so it is not the best choice for image fusion method. To this end, many researchers have done more in-depth research. After contourlet transform, Cunha et al. [11] proposed a multiscale decomposition method superior to contourlet transform, which is an improvement of contourlet transform, called nonsubsampled contourlet transform. NSCT has the characteristics of translation invariance and avoiding spectral aliasing. The structural information of the source image is preserved in the decomposition and reconstruction, and the direction information can be better extracted. Nonsubsampled contourlet transform is one of methods widely used in medical image fusion of transform domain in recent years. Firstly, the source image is decomposed by NSCT to obtain the coarse layer and the detailed layer, and then, the multiscale and multidirection decompositions are calculated by NSPFB and NSDFB filters to obtain subband images with different scales and directions.

Coarse layer fusion with low-frequency band fusion rules:

(1) $$\begin{array}{c} L_{k}^{F}\left(p\right)=\left\{\begin{array}{cc} L_{K}^{A}\left(p\right), & \text{if }u_{K}^{A}\left(p\right)\geq u_{K}^{B}\left(p\right),\\ L_{K}^{B}\left(p\right), & \text{if }u_{K}^{A}\left(p\right)<u_{K}^{B}\left(p\right). \end{array}\right. \end{array}$$

Fusion detail layer with high-frequency band fusion rules:

(2) $$\begin{array}{c} D_{k,h}^{F}\left(p\right)=\left\{\begin{array}{cc} D_{K,h}^{A}\left(p\right), & \text{if }g_{K,h}^{A^{2}}\left(p\right)\geq g_{K,h}^{B^{2}}\left(p\right),\\ D_{K,h}^{B}\left(p\right), & \text{if }g_{K,h}^{A^{2}}\left(p\right)<g_{K,h}^{B^{2}}\left(p\right). \end{array}\right. \end{array}$$

**Figure 3:**
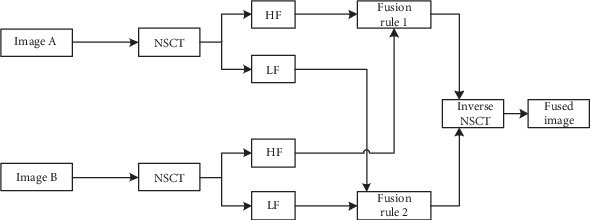
Framework diagram based on the NSCT domain fusion method.

Finally, the image is inverse NSCT to obtain a fused image. A block diagram of the NSCT-based fusion method is shown in Figure 3.

Most algorithms will do in-depth research and changes on the fusion rules.

The rules for merging high-frequency subbands are Log-Gabor, local energy-based weighting strategy, type-2 fuzzy logic algorithm [12], and adaptive two-channel pulse-coupled neural network algorithm (PCNN) [13], based on improved PCNN (IPCNN) [14] and significant matching measure rules [15].

The rules for merging low frequency subbands are phase consistency, weighting strategy based on gray mean deviation, local energy algorithm based on local features [12], sparse representation algorithm (SR) [13, 15], and based on improved PCNN (IPCNN) [14].

In order to solve quality problem of fused images, Xinqiang et al. [16] proposed an image fusion method based on local neighborhood features and NSCT. Firstly, NSCT processing is performed on the source image to obtain LF and HF in each direction, where a weighted fusion strategy based on gray mean deviation is used for LF, a weighted fusion strategy based on local energy is used for HF, and a fused image is transformed by inverse NSCT. In order to extract more useful feature information, Padmavathi et al. [17] proposed a new fusion method which combines Darwinian particle swarm optimization algorithm with NSCT. Elements in particle swarm optimization (PSO) can be used to extract the required features and remove redundant parts [18]. It is a good way to extract features. However, the shortcoming of PSO algorithm is that elements may be fixed on incorrect local optimal points. Darwinian particle swarm optimization algorithm is proposed to solve the shortcoming by DPO. The fusion image effect obtained by NSCT+DPSO algorithm is better than that of PSO, and the storage requirement is lower. Mohammed et al. [13] proposed a multimodal combination method which is based on the NCST, in which the sparse representation algorithm is used to fuse the low-frequency band, and the high-frequency band is fused by the adaptive two-channel pulse-coupled neural network. The fusion image quality of this method is high and can be captured. The fusion image of this method has high quality, can capture subtle details, adapts to the characteristics of HVS, and shows good performance in both objective and subjective analysis. Because the method uses SR and PCNN algorithms, it causes large computational defects. Tian et al. [14] proposed an improved PCNN (IPCNN) multimodal medical image fusion algorithm based on the NSCT domain. In the traditional PCNN model, the local regional singular value was introduced as the connection strength parameter of the neurons in the PCNN model to construct the local structural information factor and activate the neurons to form the improved PCNN model. The model is used to fuse high- and low-frequency coefficients, and the fused image has better robustness, reliability, and visual effects. Recent research has emerged a fusion algorithm combining NSCT-based PCNN and shuffled frog leaping algorithm, which significantly improves the spatial resolution [19]. Shabanzade and Ghassemian [15] proposed that in the multimodal fusion method based on NSCT, the sparse representation algorithm is used to fuse the low-frequency bands, and how to select a good dictionary is the key to the sparse representation algorithm. Therefore, a dictionary learning algorithm based on the combination of principal component analysis and clustering method is proposed. It can effectively separate the significant characteristics of the low-pass band coefficient; overcome the shortcomings such as the slow speed of K-SVD computer, the limitation of DCT basis, or wavelet basis by input image; and has the advantages of fast computing speed, low cost, compact structure, and strong adaptability. At the same time, the high-frequency subband is fused by the explicit matching measure rule. This method is superior to the multiscale transform and sparse representation based on visual effects and quantitative indicators.

However, some researchers tend to combine NSCT and other algorithms into new methods.

Madanala and Jhansi Rani [6] combined the advantages of frequency and time localization of wavelet transform and displacement invariance of nonsubsampled contourlet transform and proposed a fusion framework based on DWC+NSCT domain cascade. In this framework, wavelet transform was used to decompose the source image in the first stage in order to obtain the detailed coefficient and approximate coefficient, and principal component analysis method was used to fuse the detailed coefficient and approximate coefficient to minimize the redundancy. Finally, inverse wavelet transform was used to obtain the reconstruction in the first stage. In the second stage, NSCT is applied to the products in the first stage to obtain the high-frequency and low-frequency coefficients. The maximum selection rule is used for fusion, and then, the final fusion image is obtained by inverse NSCT. The second stage solves the displacement variance problem generated in the first stage, which makes the fused image has the characteristics of strong applicability and good effect. Similarly, Bhateja et al. [20] cascaded the combination of stationary wavelet transform and nonsubsampled contour transform domain. This algorithm reduces the redundancy of fused images and enhances the contrast of diagnostic features.

#### 2.2.2. Fusion Method Based on Nonsubsampled Shearlet Transform (NSST) Domain

**Figure 4:**
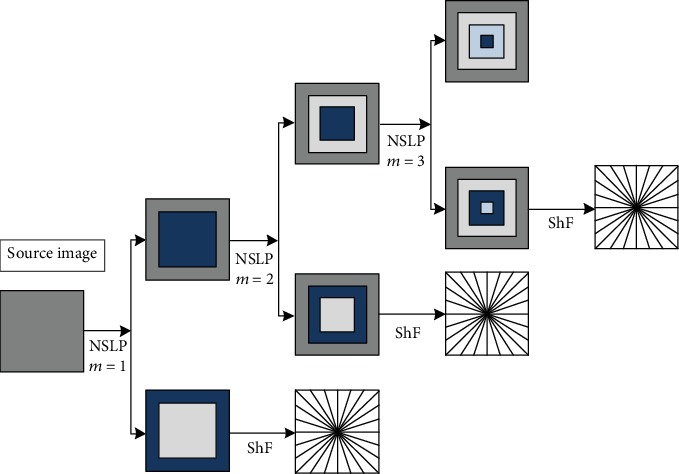
NSST diagram.

In 2005, the tool shearlet proposed by Labate et al. [21] has multiscale, directional, and other characteristic but does not have translation invariance. Until 2007, Easley et al. [22] proposed a nonsubsampled shearlet transform, which solves the problem of translation invariance on the basis of retaining the directivity of shearlets. NSST consists of a Nonsubsampled Laplacian Pyramid (NSLP) and Multiple Shear Filters. The source image is decomposed into high-frequency components and low-frequency components by NSLP, and then, the direction filter is used to process different subbands and coefficients in different directions, among which the low-frequency subband is iterative decomposition. Directional filtering is performed using a shear matrix, so it has a strong directivity. As shown in Figure 4, when the decomposition level is *m* = 3, the image is decomposed into four subbands with *m* + 1 = 4, the size of which is the same as that of the source image, thus ensuring the invariance of displacement [23]. Compared with NSCT, NSST has higher sensitivity and lower computational complexity, while overcoming the limitations of components with a certain number of directions.

#### 2.2.3. Pulse-Coupled Neural Network (PCNN) Fusion Method Based on NSST Domain

NSST is a popular transformation that highlights feature information in medical image fusion. It is often favored by researchers because of its high sensitivity, multidirectionality, and high-speed processing capability. The pixel points generated by the NSST decomposition of the source image correspond to edge information and texture information with large transform coefficients, and the amount of multimodal medical image information acquired by different sensors is greatly different. The fusion image should not only keep the characteristic information of the source image but also to ensure good visual effect and less distortion, therefore to use PCNN fusion image decomposed high-frequency coefficient [24]. PCNN is a biologically inspired feedback neural network, which is a single-layer two-dimensional horizontally connected neuron array. The PCNN neurons are composed of a receiving field (dendritic), a connected modulation field, and a pulse generator. PCNN can be used to extract useful information from source images without training process, and the characteristics of neurons make it have greater advantage in biological background. It has been widely used in image processing field [25]. However, PCNN also has many defects such as too many parameters and difficulty in setting parameters. Therefore, researchers have proposed more optimization methods.

Yin et al. [26] proposed the PA-PCNN multimodal medical image fusion method based on NSST, which decomposed the multimodal source image NSST and obtained the multiscale and multidirection representation of the source image. A new fusion strategy is proposed to fuse the low-frequency coefficients. The activity-level metric defined as WLE in the strategy solves the energy preservation problem in the image fusion processing. In order to fully extract the details in the source image, a new activity level metric WSEML weighted sum was introduced. The parameter adaptive pulse-coupled neural network (PA-PCNN) model is used to fuse high-frequency coefficients, which solves the problem of difficult parameter setting in the traditional PCNN model. Finally, NSST reconstruction is performed. The algorithm has fast convergence speed, few iterations, and good effect. It is the first example applied to medical image fusion. Ouerghi et al. [27] proposed a simplified pulse-coupled neural network (S-PCNN) based on NSST. Unlike other fusion methods, this method converts PET images into YIQ components. The NSST transform only performed for MRI images and the Y components of PET images. The low-frequency subband is fused with the standard deviation of the weight region and the local energy. The high frequency is fused by the S-PCNN with the adaptive connection strength coefficient excitation. The fusion effect of this algorithm is better, but the application range is relatively narrow. There are still many researches on PCNN fusion methods based on NSST domain [28–30].

#### 2.2.4. Frei-Chen Operator Medical Image Fusion Method Based on NSST Domain

Extracting the direction information of an image is a challenge of image fusion, and the Frei-Chen operator can obtain edge and direction information in the source image. The source image is scaled by the averaging filter to obtain 9 subgraphs, where W1-W4 is the edge subspace map, W5-W6 is the straight line, W7-W8 is the discrete Laplace transform, W5-W8 is the line subgraph, and W9 is the average of 9 subgraphs.

After Mishra et al. [31] used the Frei-Chen operator for infrared and visible image fusion to achieve good results, Ganasala [32] proposed a Frei-Chen operator medical image fusion method based on NSST domain. Similar to other methods, NSST decomposition is carried out on the source image, but in order to preserve the significance features and maintain the structural similarity of the source image, Frei-Chen operator is used to define the appropriate significance or activity measurement for the approximate subband and detail subband coefficients, and the coefficient is selected according to its measuring value. The quality standard values of image fusion in different data sets are better, and the quantitative evaluation indicators are superior to the existing methods. There are still many new algorithms based on the combination of the NSST domain and other algorithms [33, 34], which is still an area of interest for many researchers.

#### 2.2.5. Fusion Method Based on Discrete Wavelet Transform (DWT)

**Figure 5:**
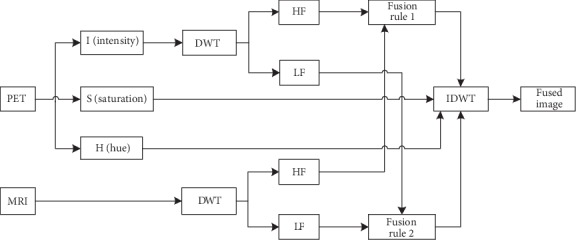
Block diagram based on the NSCT fusion method.

Discrete wavelet transform can make different input frequency signals maintaining stable output and has good positioning in the time domain and frequency domain, which helps to preserve the specific information of the image. Therefore, discrete wavelet transform (DWT) is the most widely used transform in the early research of multimodal medical image fusion algorithms. The discrete wavelet transform overcomes the limitations of the principle component analysis and has a good visual and quantitative fusion effect. Most of the DWT-based fusion methods are applied to MRI and PET image fusion [35, 36] but also to others [37]. The source image is preprocessed and enhanced, and the intensity component is extracted from the PET image using the IHS transform, which preserves more anatomical information and reduces color distortion. The DWT transform is performed on the intensity components of MRI and PET to obtain high- and low-frequency subbands. The high- and low-frequency subbands are, respectively, fused by different fusion rules, and the inverse DWT transform is performed to obtain the fused image [38]. A block diagram based on the DWT fusion method is shown in Figure 5.

Most of the researchers have in-depth research on the fusion rules. Different fusion rules show different fusion effects. The fusion rule 1: the average method [35, 38]; fusion rule 2: fuzzy—c means clustering [35]. In view of the shortcomings of discrete wavelet transform without displacement invariance and no phase information, the researchers introduced complex wavelet transform (CWT) [39]. On the basis of complex wavelet transform, Singh et al. [40] proposed a multimodal medical image fusion method based on Daubechies complex wavelet transform (DCxWT), which is superior to the spatial domain fusion method (PCA and linear fusion) and discrete wavelet method in transform domain. The dual-tree complex wavelet transform (DTCWT) proposed by Kingsbury [41] has directional selectivity and displacement invariance and can preserve the edge details of the source image. It is also an effective image fusion method [42, 43], but in image decomposition, the factors that are affected by the direction are relatively large. In recent years, researchers have often combined DTCWT with other algorithms to form new methods.

Padmavathi et al. [44] proposed a method based on the combination of dual-tree complex wavelet transform and principal component analysis (DTCWT-PCA). The principal component analysis method is one of the multivariate analysis methods based on eigenvectors. It is better to remove redundant information generated by DTCWT decomposition, and it is also the direction of block-level fusion development. Talbi and Kholladi [45] proposed a hybrid algorithm based on dual-tree complex wavelet transform and predator-prey optimizer (DTCWT+PPO), combining DTCWT and PPO, and using the mutual information technology to obtain the dual advantages of the two methods. The absolute high-value method is used to fuse the decomposed high-frequency coefficients, the weighted average method is used to fuse the low-frequency coefficients, the predator-optimizer is used to estimate and optimize the weights, and finally, the inverse transform is used to obtain the fused images. The algorithm is characterized by high robustness and high efficiency.

### 2.3. Image Fusion Based on Deep Learning

Deep learning is a new field of medical image fusion research in recent years. Convolutional neural network (CNN) is a typical deep learning model proposed by Krizhevsky et al. [46]. Compared with medical image fusion, deep learning is widely used in the segmentation of medical images [47–49] and registration of medical images [50–52]. The medical image fusion methods based on spatial domain and transform domain have the defects of activity level measurement (feature extraction) and fusion rules, which need artificial design, and the correlation between them is extremely small. In order to overcome the above problems, Liu et al. [53] applied CNN to image fusion for the first time in 2017, achieving good results relative to the spatial domain and the transform domain. The U-Net network model is widely used in medical image segmentation. From 2D to 3D [54, 55], its research technology has been relatively mature and has achieved good results in the field of medical image segmentation, but medical image fusion is a new field.

CNN is a multistage feedforward artificial neural network with trainable supervised learning. The convolution operation is multidimensional. In a convolutional network, the first parameter is usually called an input, and the second parameter is called a kernel function, and the output is called a feature map. Sparse representations (also known as sparse weights), parameter sharing, and isomorphic representations are three important architectural ideas of CNN. Traditional neural networks use matrix multiplication to deal with connection relationships. An output unit is associated with each input unit, which inevitably requires a lot of storage. However, the nature of the sparse representation of the convolutional network and the neurons are only connected to several neurons adjacent to the previous stage, and the local convolution operation is performed, which reduces the storage requirements and improves the computational efficiency. CNN's parameter sharing abandons the nonuniqueness of weights in traditional networks. The weights in the CNN stage are constant, which is better than others in storage requirements. Traditional automatic encoders are fully connected. Vector output and source image are not necessarily aligned in space, while U-Net uses local connection structure. Vector output and source image are aligned in space, so the visual effect of fusion image is better. U-Net is a full-convolution network [56], which consists of contraction path and expansion path. In-depth learning training needs a large number of samples, while U-Net is improved based on full convolution neural network, and can train a small number of samples using data enhancement. This advantage just caters to the shortcoming of a small sample size of medical image data.

#### 2.3.1. Image Fusion Method Based on Convolutional Neural Network (CNN)

**Figure 6:**
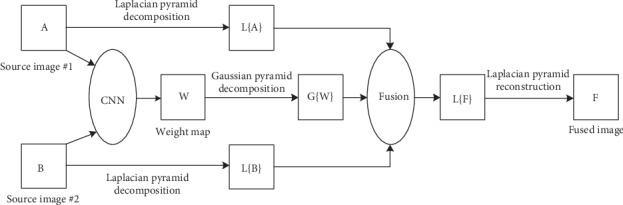
Schematic diagram based on CNN fusion algorithm.

Medical images differ in intensity at the same location, so the fusion method proposed in [53] is not suitable for medical image fusion. Yu et al. [57] first proposed a medical image fusion method based on CNN. This method uses the siamese network to generate weight map. The siamese network [58] is one of the three models for comparing patch similarity in the CNN model. Because its two weight branches are the same, the feature extraction or activity level measurement methods of the source image are the same. This has certain advantages over the models of pseudosiamese and 2-channel, and the ease of training of the siamese model is also the reason why it is favored in fusion applications. After obtaining the weight map, the Gaussian pyramid decomposition is used, and the pyramid transform is used for multiscale decomposition, so that the fusion process is more in line with human visual perception. In addition, the localized similarity-based fusion strategy is used to adaptively adjust the decomposed coefficients. The algorithm combines the common pyramid-based and similarity-based fusion algorithm with the CNN model to produce a superior fusion method.  Figure 6 is a model of the algorithm.

CNN is a new challenge in the medical field; the main reasons are (a) a large number of annotated training set data is required, (b) training takes a long time, and (c) the convergence problem is complicated, and the overfitting needs to be adjusted repeatedly. As for the problem that a large number of annotated training sets are needed, Liang et al. [59] proposed that the MCFNet network method refers to different forms of medical image histograms and transforms 1.2 million natural images in ILSVRC 2013 ImageNet into medical images with similar intensity or texture distribution as training data sets. Reconstructed data sets are very similar to medical image data sets. In order to avoid overfitting, 256∗256 images are randomly extracted from the transformed images and trained with medical images. The optimization of the loss function of this method is still the direction of future research. Following Liu, Hermessi et al. [60] proposed the CNN+shearlet fusion method to achieve a good fusion effect. Using the full convolution siamese architecture, the training framework is the famous MatConvNet. It can well retain information, and visual perception is better than the CNN+MF method. However, there are some problems such as long training time and difficult architecture. This is also a direction for future research in this field. Vu et al. [61] proposed a fusion method based on the combination of sparse self-encoder and convolutional neural network. The preprocessing SAE was added to the CNN classifier, which is better than the previous CNN. The fusion method based on CNN began to develop [62–64].

#### 2.3.2. Image Fusion Method Based on U-Net

The existing medical image fusion methods neglect the image semantics, do not pay attention to the processing of semantic conflicts, and lose useful semantic information. As a result, the fused image appears blurred boundary, which makes it more difficult for medical workers to parse the fused image. Fan et al. [65] proposed a medical image fusion method based on semantics, which solved the problem of semantic loss of fused images. In this algorithm, two U-Nets are used to construct the FW-Net network model. It is not the first time to combine U-Net with an automatic encoder in medical image research [66]. The left and right structures of FW-Net are the encoder and the decoder. They both follow the structure of U-Net. The encoder is used to extract the semantics of the source image, and the decoder is used to reconstruct the source image. FW-Net can extract the semantics of brightness in the source image and then automatically maps the brightness of different modal images to the same semantic space for image fusion. In order to obtain a smooth and clear image, bilinear interpolation is added to each layer of the encoder and decoder in FW-Net framework. There is no semantic conflict in the fused image, which is superior to other methods in visual effect. This algorithm is only applied to MRI and CT. Other pattern fusion, such as MR and PET fusion and MR and SPECT fusion, will be a research trend in the future. At the same time, the research of U-Net in medical image is not yet mature, so the research of U-Net in medical image fusion is also a focus.

## 3. The Way of Multimodal Fusion

MRI, also known as Magnetic Resonance Imaging, provides information on the soft tissue structure of the brain without functional information. The density of protons in the nervous system, fat, soft tissue, and articular cartilage lesions is large, so the image is particularly clear and does not produce artifacts. It has a high spatial resolution and no radiation damage to the human body, and the advantage of rich information makes it an important position in clinical diagnosis. The density of protons in the bone is very low, so the bone image of MRI is not clear. The CT image is called Computed Tomography imaging. The X-ray is used to scan the human body. The high-density absorption rate of bone tissue relative to soft tissue makes the bone tissue of the CT image particularly clear. The low permeability of X-rays in soft tissue leads to low absorption rate, so CT images show less cartilage information, which represents anatomical information. SPECT is called Single-Photon Emission Computed Tomography, which is a functional image that displays the metabolism of human tissues and organs and the blood flow of arteries and veins. It provides good and malignant information of tumors and is widely used in the diagnosis of various tumor diseases. However, the resolution of SPECT is low and the positioning ability is poor. The PET image is called Positron Emission Tomography, which reveals the true information of blood flow and can accurately identify the location of the patient's lesion. Its principle is using positrons to generate *γ* photons in collision with electrons in the tissue. The purpose of PET is to detect the number of *γ* photons, showing a color image of brain function information, suitable for tumor detection; its sensitivity is high, but it is difficult to obtain accurate brain structure position information; soft tissue and bone boundary resolution is lacking, so the spatial resolution is very low and the spatial distortion is highly probable.

There are many fusions of imaging methods in medical image fusion, such as MRI and PET, MRI and CT, MRI and SPECT, CT and PET, CT and SPECT, SPECT and PET, and MRI-T1 and MRI-T2. Different ways of integration keep their own characteristics, such as MRI/PET fusion images which are important for detecting liver metastasis, Alzheimer's disease, and brain tumor diagnosis; MRI/SPECT fusion images are helpful for the localization of lesions and vertebral bone metastasis in tinnitus patients; CT/PET fusion image energy improves the diagnosis of lung cancer; SPECT/PET for abdominal research; and ultrasound/MRI for vascular blood flow diagnosis. The following will focus on a few hot ways of fusion.

### 3.1. MRI and PET Fusion

**Figure 7:**
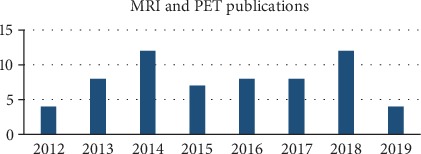
MRI/PET fusion research trend chart.

MRI is a gray image while PET is a color image, which is easily distorted in the fusion processing. In most fusion algorithms, the IHS model is used to decompose the intensity components of PET image [8], and BEMD, Log-Gabor transform, and other algorithms are combined to process these components, so as to preserve more color of PET image. Yin et al. [26] proposed an MRI and PET image fusion algorithm based on NSST and S_PCNN, which converts the PET image into YIQ component, and then used NSST to decompose MRI and the Y component of PET into low-frequency and high-frequency subbands. The simplified PCNN model was used to process high-frequency coefficients; the fused image has good effect, small color distortion, and rich structural information. Wang et al. [37] proposed a preparation method based on discrete wavelet transform for preprocessing of MRI and PET image fusion, which solved the problem of quality degradation and unreadability of input images, and the fusion accuracy was as high as 90%-95%. Chaitanya et al. [67] proposed a new fusion method by combining shearlet transformation and discrete cosine transform. Arash and Javad [68] first applied adaptive filters to the fusion of MRI-PET images, using spatial and spectral difference criteria to optimize filter coefficients. There are other MRI/PET fusion methods [69–71]. MRI/PET images are often involved in the clinical diagnosis of Alzheimer's disease, and the fusion of MRI and PET images is what is needed to meet the diagnosis. MRI/PET is a key element in tumor diagnosis. In the near future, PET/MRI may emerge as a powerful multimodal technique in clinical oncology.  Figure 7 shows the trend of articles related to MRI/PET fusion research in recent years (statistical time is from 2012 to August 2019).

### 3.2. MRI and CT Fusion

**Figure 8:**
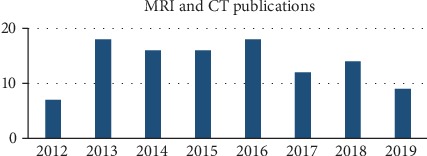
MRI/CT fusion research trend chart.

The combination of MRI and CT combines the advantages of clear bone information in CT images and the clear soft tissue of MRI images to compensate for the lack of information in a single imaging. Na et al. [72] proposed a MRI and CT fusion algorithm based on guided filtering (GF). The fused image not only preserves the edge information of the source image but also extracts the feature information, which solves the problem of edge degree and clarity. In [31], the Frei-Chen operator fusion algorithm based on NSST domain is proposed. The visual analysis of fusion results has obvious improvement in contrast and structural similarity. Quantitative evaluation is also a further improvement of existing methods. In [73], the membership degree is difficult to select based on the intuitionistic fuzzy inference fusion algorithm. Mishra et al. [31] further proposed the fuzzy-PCNN rule, using multiple membership functions to generate fuzzy membership from specific parts of high-frequency coefficients; L2 norm set operation is applied to the results, using the rule to fuse the high-frequency coefficient; SF, EN, and SD of fused image have a higher value. Following [74] fusion of MRI/CT images in the NSST domain, Singh et al. [33] proposed a new fusion method using the ripple transform and NSST transform cascade, which has a good effect on visual quality and quantitative indicators. Other methods for MRI/CT image fusion include contourlet transform based on non-sub-sampling [75] and multiscale and multiresolution methods [76].  Figure 8 shows the trend of articles related to MRI/CT fusion studies in recent years (the statistical time is from 2012 to August 2019).

### 3.3. MRI and SPECT Fusion

**Figure 9:**
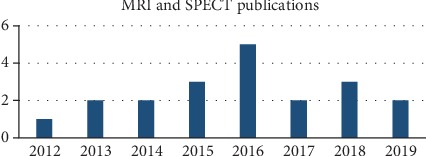
MRI/SPECT fusion study trend chart.

The fusion image of MRI and SPECT has both functional information and structural information, which greatly helps noninvasive diagnosis. In order to extract the salient features of the image, Shahdoosti and Tabatabaei [77] proposed a new fusion algorithm by combining the antolony algorithm with the integrated empirical mode decomposition domain (EEMD), which provides a lot of spatial and color information. Du et al. [78] proposed the fusion method of anatomical image and functional image fusion using parallel significant features. Different significant features were utilized in image fusion such as MRI-CBV/SPECT-Tc and MRI-T1/SPECT-FDG. Fusion images could retain edge information in anatomical image and color detail information in functional image, with advantages of high spatial resolution and high intensity. In the fusion method based on NSST domain proposed by Xiong et al. [25], PA-PCNN and the new fusion strategy were used to fuse the high- and low-frequency subbands. The algorithm is superior to NSCT+SF+PCNN, SR+SOMP, GF, and NSCT+PCPD in terms of color preservation, and it is better than the NSCT+RPCNN method in detail preservation. It is an effective MRI/SPECT image fusion method. Daniel [79] proposed a homomorphic wavelet fusion method based on hybrid genetic-gray optimization algorithm, which is superior to DCTDWT FFT in mutual information, semaphore, and edge information. A fixed proportion of segmentation is prone to color distortion; Jing et al. [80] in this paper proposes a new adaptive decomposition algorithm for this problem to distinguish between low frequency and high frequency which can keep color and structural information; the algorithm will be sparse representation (SR), and the edge filter is applied to image fusion, in the aspect of color information and spatial information retained superior to other methods.  Figure 9 shows the trend of articles related to MRI/SPECT fusion studies in recent years (statistical time is from 2012 to August 2019).

## 4. Comparative Analysis

### 4.1. Experimental Data Set

Most of the experimental images of medical image fusion come from the whole brain Atlas database (http://www.med.harvard.edu/aanlib/home.html). The whole brain Atlas is a benchmark database for evaluating the performance of multimodal medical image fusion methods developed by Keith A. Johnson of Harvard Medical School and MIT Journal Alex Becker. The database contains various data such as normal brain, cerebrovascular disease, neoplastic disease, recessive disease, inflammatory disease, or infectious disease. A few original images are also can be found at http://www.bic.mni.mcgill.ca/brainweb/.

### 4.2. Performance Analysis

Image fusion quality needs to be measured by a consistently accepted standard. These objective evaluation indicators are EN (entropy), MI (mutual information), standard deviation (SD), peak signal to noise ratio (PSNR), structural similarity index measure(SSIM), average gradient (AG), *Q*^*AB*/*F*^metric, root mean square error (RMSE), edge intensity (ES), visual information fidelity (VIF), spatial frequency (SF), etc. A few common evaluation indicators are as follows.

#### 4.2.1. Entropy (EN)

Entropy describes the content of the information in the image. It is a measure of the amount of information contained in an image, taking values between 0 and 8. The formula is as follows:

(3) $$\begin{array}{c} \text{EN}=-\overset{L-1}{\underset{L=0}{\sum }}p_{i}\times \log_{2}p_{i}, \end{array}$$

where *L* represents the number of gray levels and is a probability density function for each gray value *i*. The entropy value is proportional to the amount of information contained in the fused image.

#### 4.2.2. Standard Deviation (STD)

The standard deviation is mainly used to measure the overall contrast of the fused image and is used to determine the difference between the data and the average. If the STD value is larger, the more useful information the fused image contains, the better the fusion effect performs, and the image is clearer. The STD calculation formula is as follows:

(4) $$\begin{array}{c} \text{STD}=\sqrt{\frac{\sum_{i=1}^{M}\sum_{j=1}^{N}{\left(f\left(i,j\right)-\mu \right)}^{2}}{MN}}, \end{array}$$

where *M* and *N* represent the length and width of the image *f*(*i*, *j*), which is generally 256. The average value of the fused image is represented by *μ*.

#### 4.2.3. Mutual Information (MI)

Mutual information is a measure of the dependence between two input source images (*X*, *Y*). Mutual information is how much information is calculated in the source image and transferred to the fused image. The mutual information is proportional to the fused message. The formula for calculating MI is as follows:

(5) $$\begin{array}{c} \text{MI}=I\left(x,\;f\right)+I\left(y,\;f\right),\\ I\left(x,y\right)=\underset{y\in Y}{\sum }\underset{x\in Y}{\sum }p\left(x,y\right)\log\frac{p\left(x,y\right)}{p\left(x\right)p\left(y\right)}, \end{array}$$

where *p*(*x*) and *p*(*y*) are the edge probability density functions of the two images, and *p*(*x*, *y*) is the joint probability density function of the fused image and the source image *X*, *Y*.

#### 4.2.4. Peak Signal to Noise Ratio (PSNR)

PSNR is a quantitative measurement method based on mean square error. In the fusion image, the higher the PSNR is, the better the SNR is, and the closer it is to the source image.

(6) $$\begin{array}{c} \text{PSNR}=10*\log_{10}\left(\frac{L^{2}}{{\text{RMSE}}^{2}}\right). \end{array}$$

It represents the maximum pixel gray value in the fused image, which is generally 255. RMSE is the mean square error, and its calculation formula is

(7) $$\begin{array}{c} \text{RMSE}=\sqrt{\frac{\sum_{m=1}^{M}\sum_{n=1}^{N}{\left[\text{ground}\left(m,n\right)-\text{fused}\left(m,n\right)\right]}^{2}}{M\times N}}. \end{array}$$

Mean square error is an image quality measurement method. The value of RMSE is inversely proportional to the quality. The lower the value of RMSE, the better the quality of the fused image have. ground(*m*, *n*) and fused(*m*, *n*) represent the intensity values of the source image and the fused image pixel, respectively, and the length and width of the image are *M* and *N*, respectively.

#### 4.2.5. Structural Similarity Index Measure (SSIM)

SSIM is a measure of the structural similarity between a fused image and a source image. Its value is between 0 and 1, with 0 indicating zero correlation with the original image and 1 indicating the exact same image. The larger the SSIM value, the more similar the fused image is to the source image; that is, the fusion effect is better.

(8) $$\begin{array}{c} {\text{SSIM}}_{\left(A,B,F\right)}=0.5\times \left({\text{SSIM}}_{\left(A,F\right)}+{\text{SSIM}}_{\left(B,F\right)}\right). \end{array}$$

In

(9) $$\begin{array}{c} {\text{SSIM}}_{\left(A,F\right)}=\frac{\left(2\mu_{A}\mu_{F}+C_{1}\right)\left(2\sigma_{AF}+C_{2}\right)}{\left(\mu_{A}^{2}+\mu_{F}^{2}+C_{1}\right)\;\left(\sigma_{A}^{2}+\sigma_{F}^{2}+C_{2}\right)},\\ \text{ SSI}{\text{M}}_{\left(B,F\right)}=\frac{\left(2\mu_{B}\mu_{F}+C_{1}\right)\left(2\sigma_{BF}+C_{2}\right)}{\left(\mu_{B}^{2}+\mu_{F}^{2}+C_{1}\right)\;\left(\sigma_{B}^{2}+\sigma_{F}^{2}+C_{2}\right)}. \end{array}$$

 *μ*_*A*_, *μ*_*B*_, and *μ*_*F*_ are the average values of the source image and the fused image, respectively; *σ*_*A*_^2^, *σ*_*B*_^2^, and *σ*_*F*_^2^ are the variances of the source image and the fused image, respectively; *σ*_*AF*_ and *σ*_*BF*_ represent the joint variance of the two source images and the fused image, respectively.

#### 4.2.6. Spatial Frequency (SF)

The spatial frequency reflects the sharpness of the fused image, that is, the rate of change of the image gray; the larger the SF is, the higher the image resolution perform.

(10) $$\begin{array}{c} \text{SF}=\sqrt{{\text{RF}}^{2}+{\text{CF}}^{2}}. \end{array}$$

In

(11) $$\begin{array}{c} \text{RF}=\sqrt{\frac{1}{M\left(N-1\right)}\overset{M}{\underset{i=1}{\sum }}\overset{N}{\underset{j=2}{\sum }}{\left(X\left(i,j-1\right)-X\left(i,j\right)\right)}^{2}},\\ \text{CF}=\sqrt{\frac{1}{\left(M-1\right)N}\overset{M}{\underset{i=2}{\sum }}\overset{N}{\underset{j=1}{\sum }}{\left(X\left(i,j\right)-X\left(i-1,j\right)\right)}^{2}}. \end{array}$$

RF and CF are the row and column frequencies of the image, respectively.

#### 4.2.7. *Q*^*AB*/*F*^ Measurement

*Q* ^*AB*/*F*^ measures the amount of edge information from the source image to the fused image through the Sobel edge detection operator. The larger the value of *Q*^*AB*/*F*^ represent, the more information is converted from the source image, and the edge information is better preserved. In general, high edge strength has a greater impact on *Q*^*AB*/*F*^ than low edge strength.

(12) $$\begin{array}{c} Q^{AB/F}=\frac{\sum_{n=1}^{N}\sum_{m=1}^{M}\left(Q^{A}\left(n,m\right)W^{A}\left(n,m\right)+Q^{B}\left(n,m\right)W^{B}\left(n,m\right)\right)}{\sum_{n=1}^{N}\sum_{m=1}^{M}\left(W^{A}\left(i,j\right)+W^{B}\left(i,j\right)\right)}, \end{array}$$

where *Q*^*A*^(*n*, *m*), *Q*^*B*^(*n*, *m*) is the edge information storage value; *W*^*A*^(*n*, *m*), *W*^*B*^(*n*, *m*) is the weighting map.

### 4.3. Experiments and Analyses

**Figure 10:**
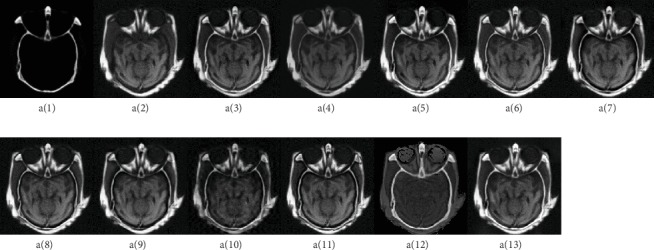
Examples of MRI-CT medical image fusion, a(1) and a(2) are CT and MRI source images, respectively. a(3)~a(13) are the fused images of GFF [81], MSA [82], NSCT+SR [83], NSCT+PCNN [84], NSCT+LE [85], NSCT+RPCNN [86], NSST+PAPCNN [26], DWT [87], DWT+WA [88], U-Net [65], and CNN [57], respectively.

**Figure 11:**
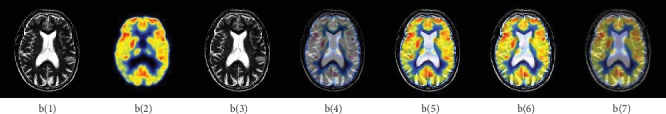
Examples of MRI-PET medical image fusion, b(1) and b(2) are MRI and PET source images, respectively. b(3)~b(7) are the fused images of GFF [81], MSA [82], NSCT+LE [85], NSST+PAPCNN [26], and ESF+CSF [78], respectively.

To compare the three classifications of the medical image fusion methods in Section 2, we performed two experiments: MRI/CT and MRI/PET. The resolution of each test image is set to 256 × 256. The images are shown in Figures 10 and 11; in Figure 10, a(1) and a(2) show CT and MRI source images, respectively, and in Figure 11, b(1) and b(2) show MRI and PET source images, respectively. Medical image pairs are obtained from http://www.med.harvard.edu/aanlib/home.html.

#### 4.3.1. Experiment and Evaluation on the Group of MRI-CT Fusion

In this section, the methods for comparison are eleven MRI-CT image fusion algorithms based on GFF [81], MSA [82], NSCT+SR [83], NSCT+PCNN [84], NSCT+LE [85], NSCT+RPCNN [86], NSST+PAPCNN [26], DWT [87], DWT+WA [88], U-Net [65], and CNN [57], respectively. From a(3) to a(13) in Figure 10 show the fused images of these fusion methods. In order to evaluate the performance of the above different multimodal medical image fusion methods, seven metrics are applied to the objective quality assessments, such as EN, STD, MI, PSNK, SSIM, SF, and *Q*^*AB*/*F*^ [89]. After that, the objective performances of those methods are shown in Table 1 (the highest value of each metrics is marked in underline).

From Table 1, the highest *Q*^*AB*/*F*^ value is the algorithm GFF, which saves the edge information of the source image better. The MSA algorithm and MSCT+SR performed best on the PSNK and STD evaluation indicators, respectively. The NSCT+PCNN algorithm performs well on the MI and SSIM evaluation indicators. The fusion image a(6) is also superior to other fusion methods in terms of visual effects, indicating that the fusion image has more useful information and is similar to the source image. The sharpness of a(11) in Figure 10 is better than other methods, so the algorithm DWT + WA has the highest value on SF. The advantage of deep learning lies in feature extraction, so the algorithm CNN achieves the best in the EN index, and the amount of information contained in the fused image is the largest.

#### 4.3.2. Experiment and Evaluation on the Group of MRI-PET Fusion

In this section, the methods for comparison are eleven MRI-PET image fusion algorithms based on GFF [81], MSA [82], NSCT+LE [85], NSST+PAPCNN [26], and ESF+CSF [78], respectively. b(3) to b(7) in Figure 11 show the fused images of these fusion methods. In order to evaluate the performance of the above different multimodal medical image fusion methods, seven metrics are applied to the objective quality assessments, such as EN, STD, MI, PSNK, SSIM, SF, and *Q*^*AB*/*F*^ [89]. After that, the objective performances of those methods are shown in Table 2 (the highest value of each metrics is marked in underline).

From Table 2, the GFF algorithm performs well on the MI, PSNR, SSIM, SF, and *Q*^*AB*/*F*^ evaluation indicators. This algorithm uses guided filtering to control the saliency and spatial consistency of pixels, thereby improving the quality of the fused image. The highest STD value is the algorithm NSST+PAPCNN. Firstly, this algorithm converts PET color images to YUV, then, fuses the Y component with MRI. It can maintain the color without distortion, and the fusion effect is very good.

## 5. Summary

The development of medical image fusion ranges from spatial domain, transform domain, to deep learning. Its rapid development also indicates a high demand of computer-aided clinical diagnosis. Different researchers propose different fusion methods, each of which has its own advantages in different evaluation indicators. However, there are nearly 30 kinds of evaluation indexes for medical image fusion. The quantitative evaluation indicators used by researchers are often different for different fusion effects. The nonuniqueness of evaluation indicators brings limitations to the application prospects. On the other hand, although the research on medical image fusion is very popular, its innovation is relatively low. Most of the fusion methods are modified based on the original methods, and the problems existing in the fusion effect are only improved but not completely solved, such as color distortion and feature information extraction. Applying innovative algorithms to medical image fusion remains a huge challenge in this area of research. Deep learning has improved the effect of fusion, but the research also has certain defects; for example, the framework of deep learning is single and the amount of data for training is small. Because the trained images require professional labeling by medical experts, the workload is large and the cost is high. Therefore, the training data is lacking, and the lack of data may lead to overfitting. Applying the feature information and the lesion information to the data set obtained by the data augmentation has a certain influence on the accuracy. Therefore, how to obtain a huge data set is a difficult point in medical image research. The training of deep learning is time-consuming, and the framework is complex, which requires high requirements for computer hardware configuration. It is an important part of its research to simplify the training model or put forward a new training model and parallel training. Partial fusion method relies on accurate image registration and has little independence.

There is a difference in medical image information obtained by different sensors. The current research hotspot is the fusion of two modes, and the fusion of the three modes is rarely studied. The two modal studies focus on the fusion of MRI/CT, MRI/PET, and MRI/SPECT. From the experiments in Tables 1 and 2, we can see that the most open source is MRI/CT, so researchers favor the research of MRI/CT fusion methods; other fusions are still a challenge. Some fusion algorithms are only for single fusion methods such as MRI/CT or MRI/PET, and the compatibility of the algorithm is relatively small. Clinical diagnostic needs are not limited to the fusion of structural and functional information images, such as thyroid tumor diagnosis needs CT, MRI, SPECT, and B-ultrasound; future fusion of multiple modes and algorithm compatibility is a challenging topic.

To sum up, this article from the medical image fusion method and different image fusion methods on medical image fusion research in recent years are discussed, combining the proposed fusion method in recent years and the advantages of different methods and fusion effect; for the way the different imaging fusion method and the research trend of statistics, this paper expounds the platform of research and data sets. According to the previous part, the research of deep learning in medical image fusion is the future trend.

## Figures and Tables

**Table 1:** Evaluation index data of different methods of MRI-CT.

	Fusion methods	MRI-CT evaluation standards						
		EN	STD	MI	PSNR	SSIM	SF	*Q* ^*AB*/*F*^
Spatial domain	GFF [81]	6.7971	53.7262	3.4313	31.1594	0.4865	16.0034	0.785
	MSA [82]	6.3804	43.5218	3.9185	35.0709	0.4829	10.8225	0.5526

Transform domain	NSCT+SR [83]	6.8938	60.7771	3.127	29.5602	0.4825	17.6378	0.7761
	NSCT+PCNN [84]	6.7391	58.8135	5.0068	31.2341	0.5043	17.0262	0.7775
	NSCT+LE [85]	6.8011	57.3437	3.2865	31.6099	0.4861	16.9265	0.7369
	NSCT+RPCNN [86]	6.7696	57.972	4.4187	31.6844	0.5002	17.2528	0.7659
	NSST+PAPCNN [26]	6.8406	57.2625	3.2309	32.9194	0.4914	15.8548	0.7059
	DWT [87]	6.5888	41.9861	1.9772	31.972	0.4293	13.3888	0.5507
	DWT+WA [88]	6.2981	55.4057	4.7547	30.9814	0.4875	18.1062	0.7772

DL	U-Net [65]	5.13	42.8826	2.2207	26.4196	0.3225	17.7593	0.3127
	CNN [57]	7.0062	60.0385	3.0757	28.9646	0.4751	17.6211	0.7747

**Table 2:** Evaluation index data of different methods of MRI-PET.

	Fusion methods	MRI-PEG evaluation standards						
		EN	STD	MI	PSNR	SSIM	SF	*Q* ^*AB*/*F*^
Spatial domain	GFF [81]	4.4312	63.882	3.127	42.9419	0.7098	31.7488	0.5978
	MSA [82]	4.5174	55.9475	2.8495	28.75	0.662	17.0158	0.3038

Transform domain	NSCT+LE [85]	4.5659	75.5464	2.6052	26.0083	0.6729	31.337	0.5184
	NSST+PAPCNN [26]	4.62	77.6748	2.6297	25.2629	0.673	31.7002	0.5206

Others	ESF+CSF [78]	4.907	60.3976	2.84	29.6479	0.6483	21.0867	0.2845
